# Moving the Needle on Outcome Measurement: A Longitudinal Analysis of Frailty Among Home-Delivered Meal Recipients

**DOI:** 10.1093/geroni/igab046.1962

**Published:** 2021-12-17

**Authors:** Lisa Juckett, Haley Oliver, Leah Bunck, Crystal Kurzen, Andrea Devier, Fannisha Page

**Affiliations:** 1 The Ohio State University, Columbus, Ohio, United States; 2 LifeCare Alliance, Columbus, Ohio, United States

## Abstract

Frailty is a complex condition highly associated with malnutrition and can lead to the devastating loss of independence among older adults. Home-delivered meals (HDMs) aim to combat frailty and malnutrition and provide nutritional support to nearly 10 million older adults each year. Though self-reported metrics indicate that HDMs help older adults maintain their independence, few studies have systematically collected longitudinal data that objectively represent the health benefits of HDMs. The present study implemented two evidence-based instruments designed to measure frailty levels of HDM recipients (age 60 to 99 years) at two time points. HDM staff at one organization underwent multifaceted training to implement The Home Care Frailty Scale and the Clinical Frailty Scale with HDM recipients at the start of HDM enrollment and at three-month follow-up. Activity of daily living impairments (B = .46, p < .001) and instrumental activity of daily living impairments (B = .28, p < .001) were significant predictors of higher frailty levels at baseline (N = 245). Sixty-two recipients were analyzed at 3-month follow-up. Clinical Frailty Scale scores indicated stable frailty levels from baseline to follow-up (4.08 vs. 4.08). Home Care Frailty Scale scores indicated a slight increase in frailty levels (7.4 vs 7.63) though not statistically significant, t(61) = -.34, p = .74. These stable frailty metrics suggest that HDMs contribute to older adults’ ability to remain living in their own homes and communities and can support the importance of increased financial investments in HDM programs at the state and national levels.

